# Metagenomic and Genomic Sequences from a Methanogenic Benzene-Degrading Consortium

**DOI:** 10.1128/mra.01342-22

**Published:** 2023-04-26

**Authors:** Courtney R. A. Toth, Olivia Molenda, Camilla Nesbø, Fei Luo, Cheryl Devine, Shen Guo, Xu Chen, Elizabeth A. Edwards

**Affiliations:** a Department of Chemical Engineering and Applied Chemistry, University of Toronto, Toronto, Ontario, Canada; University of Southern California

## Abstract

Draft and complete metagenome assembled genomes (MAGs) were created from multiple metagenomic assemblies of DGG-B, a strictly anaerobic, stable mixed microbial consortium that degrades benzene completely to methane and CO_2_. Our objective was to obtain closed genome sequences of benzene-fermenting bacteria to enable the elucidation of their elusive anaerobic benzene degradation pathway.

## ANNOUNCEMENT

DGG-B refers to a scaled-up lineage (>100 L) of a methanogenic benzene-degrading enrichment culture called “OR” derived from microcosms constructed in 1995 with sediments from an oil refinery (1, 2). OR and DGG-B cultures are grown routinely in a minimal medium amended monthly with 25 mg/L benzene. In 2010, preliminary metagenomic sequencing (3) indicated that OR was composed primarily of two uncultured strains of benzene-fermenting Deltaproteobacteria (now *Desulfobacterota*), namely, ORM2a and ORM2b; a candidate phylum bacterium (OD1, later reclassified as *Candidatus* Nealsonbacteria); and two methanogens (*Methanothrix* and *Methanoregula*). OR and DGG-B also contain >100 low-abundance microorganisms that are not well characterized (2–4). To learn more about these complex microbial communities and to shed light on their unknown mechanism of benzene degradation, metagenomic assemblies were used to reconstruct draft and complete genome sequences.

**FIG 1:**
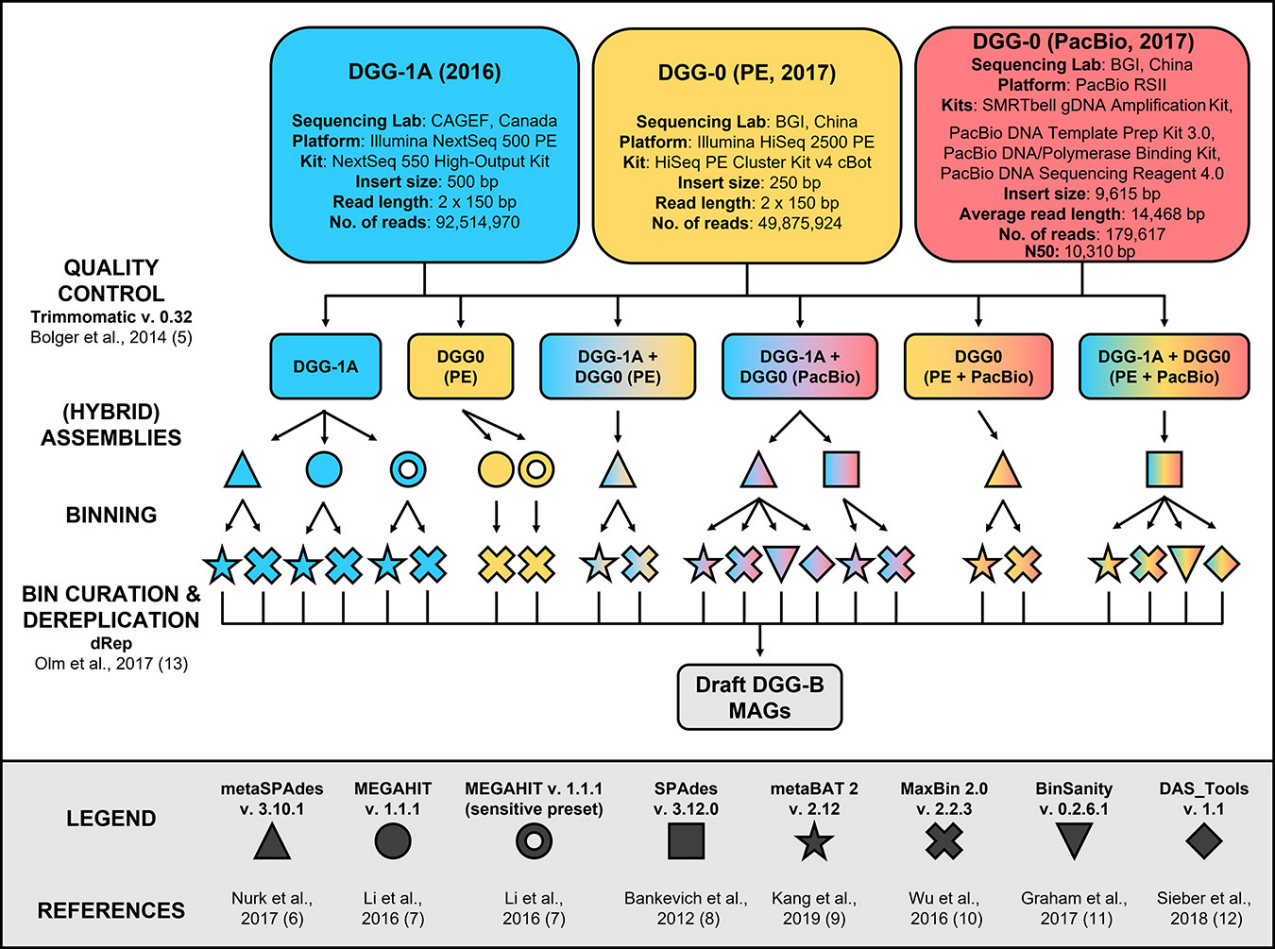
Metagenomics workflow used for (hybrid) assembly, binning, and curation of draft DGG-B MAGs. Default parameters were used for all software unless otherwise specified. PE, paired-end.

DNA was extracted from aliquots of two subcultures of DGG-B in 2016 (DGG1A, 15 mL) and in 2017 (DGG0, 200 mL) using the Qiagen DNeasy PowerSoil kit. At the time of sampling, the total volumes of DGG1A and DGG0 were 15.9 L and 9.0 L, respectively, and degraded benzene at a rate of 0.8 to 1.5 mg/L/day. Metagenomic sequencing was performed using long-read (PacBio) and/or short-read (Illumina) methods summarized in Fig. 1. Following the metagenomics workflow illustrated in Fig. 1, raw reads were trimmed and filtered (5), assembled into contigs (6–8), and binned (9–12). Default parameters were used for all software; for MEGAHIT assemblies, we also used the “sensitive” preset option. Bins with a pairwise average nucleotide identity of ≥99%, genome completeness of >50%, and contamination of <25% were compared and dereplicated (13). Taxonomy was assigned to the resulting draft MAGs using the GTDB-tk tool kit (14). Three draft MAGs were selected for further refinement (Table 1). In ABySS v.1.3.7 (15), new assemblies were created using a coverage of ≥80 (for ORM2a only) and/or *k*-mer of 96. ABySS contigs were then mapped onto the existing contigs, and gaps were resolved using a perl script published in Text S1 of Tang et al. (16). Finished genomes were polished by read mapping raw reads onto circular genomes using the Bowtie v.7.2.1 plugin within Geneious v.8.1.8 (17). Assembly information and MAG statistics were generated in CheckM v.1.0.18 (18) and using the anvi-summarize command within Anvi’o v.6.2 (19).

**TABLE 1:** Summary of two metagenomes and three closed MAGs reported in this study^*a*^

Name	Genome affiliation (GTDB-tk)	Genome affiliation (NCBI)	(Meta) genome length (Mb)	No. of contigs	GC content (%)	Avg coverage depth (×)	Genome completeness (%)	Genome contamination (%)	GenBank accession no.	IMG ID
DGG-1A metagenome	N/A^*b*^	N/A	256	191,164	51.2	56	N/A	N/A	JARDUI000000000	3300019861
DGG0 metagenome (PE + PacBio hybrid assembly)	N/A	N/A	231	355,455	49.6	28	N/A	N/A	JARDUJ000000000	3300028923
*Desulfobacterota* candidate ORM2a	*Desulfobacterota*; BSN033; UBA8473	*Desulfobacterota*	3.30	1	44.1	1,765 (DGG1A); 979 (DGG0)	100	0	CP113000.1	2795385393
*Candidatus* Nealsonbacteria DGGOD1	*Patescibacteria*; *Paceibacterales*; UBA5738	*Patescibacteria*; *Candidatus* Nealsonbacteria	1.16	1	45.8	278 (DGG1A); 113 (DGG0)	100	0	CP092821.1	2791354853
*Methanoregula* sp. strain DGG0	*Methanoregulaceae*	*Methanoregula*	2.17	1	52.0	169 (DGG1A); 78 (DGG0)	100	0	CP112999.1	2799112206

a A table summarizing all other (draft) MAGs created in this study is available in figshare (https://doi.org/10.6084/m9.figshare.21663302.v1).

b N/A, not available.

Seventy-four draft MAGs were recovered from DGG-B metagenomes, representing 71 to 73% of total microorganisms as determined by relative abundance estimates in Anvi’o (19). Further refinement enabled us to close the genomes of ORM2a, a *Candidatus* Nealsonbacteria (designated DGGOD1a), and a *Methanoregula* sp. strain (Table 1). Other MAGs recovered included members of the *Bacteroidales* (*n* = 10), *Anaerolineales* (*n* = 6), *Syntrophales* (*n* = 5), and *Methanothrix* (*n* = 8). Our laboratory is currently analyzing the genomes of ORM2a (C. R. A. Toth, O. Molenda, C. Nesbo, F. Luo, C. Devine, R. Flick, and E. A. Edwards, unpublished data) and *Ca.* Nealsonbacteria (20).

### Data availability.

Nucleotide accession numbers for reported metagenomes and refined MAGs are provided in Table 1. FASTA files and relevant statistics for all other draft MAGs are available in FigShare (https://doi.org/10.6084/m9.figshare.21663302.v1). An assembly of the original OR metagenome and a draft ORM2 bin are also available in the US DOE Joint Genome Institute Integrated Microbial Genomes (IMG) system (identifiers [IDs] 3300001389 and 2739367767, respectively).

## References

[1] Nales M, Butler BJ, Edwards EA. 1998. Anaerobic benzene biodegradation: a microcosm survey. Bioremediat J 2:125–144. doi:10.1080/10889869891214268.

[2] Toth CRA, Luo F, Bawa N, Webb J, Guo S, Dworatzek S, Edwards EA. 2021. Anaerobic benzene biodegradation linked to the growth of highly specific bacterial clades. Environ Sci Technol 55:7970–7980. doi:10.1021/acs.est.1c00508. 34041904

[3] Devine CE. 2013. Identification of key organisms, genes and pathways in benzene-degrading methanogenic cultures. PhD dissertation. University of Toronto, Toronto, Ontario, Canada.

[4] Luo F, Devine CE, Edwards EA. 2016. Cultivating microbial dark matter in benzene-degrading methanogenic consortia. Environ Microbiol 18:2923–2936. doi:10.1111/1462-2920.13121. 26549712

[5] Bolger AM, Lohse M, Usadel B. 2014. Trimmomatic: a flexible trimmer for Illumina sequence data. Bioinformatics 30:2114–2120. doi:10.1093/bioinformatics/btu170. 24695404 PMC4103590

[6] Nurk S, Meleshko D, Korobeynikov A, Pevzner PA. 2017. metaSPAdes: a new versatile metagenomic assembler. Genome Res 27:824–834. doi:10.1101/gr.213959.116. 28298430 PMC5411777

[7] Li D, Luo R, Liu CM, Leung CM, Ting HF, Sadakane K, Yamashita H, Lam TW. 2016. MEGAHIT v1.0: a fast and scalable metagenome assembler driven by advanced methodologies and community practices. Methods 102:3–11. doi:10.1016/j.ymeth.2016.02.020. 27012178

[8] Bankevich A, Nurk S, Antipov D, Gurevich AA, Dvorkin M, Kulikov AS, Lesin VM, Nikolenko SI, Pham S, Prjibelski AD, Pyshkin AV, Sirotkin AV, Vyahhi N, Tesler G, Alekseyev MA, Pevzner PA. 2012. SPAdes: a new genome assembly algorithm and its applications to single-cell sequencing. J Comput Biol 19:455–477. doi:10.1089/cmb.2012.0021. 22506599 PMC3342519

[9] Kang DD, Li F, Kirton E, Thomas A, Egan R, An H, Wang Z. 2019. MetaBAT 2: an adaptive binning algorithm for robust and efficient genome reconstruction from metagenome assemblies. PeerJ 7:e7359. doi:10.7717/peerj.7359. 31388474 PMC6662567

[10] Wu YW, Simmons BA, Singer SW. 2016. MaxBin 2.0: an automated binning algorithm to recover genomes from multiple metagenomic datasets. Bioinformatics 32:605–607. doi:10.1093/bioinformatics/btv638. 26515820

[11] Graham ED, Heidelberg JF, Tully BJ. 2017. BinSanity: unsupervised clustering of environmental microbial assemblies using coverage and affinity propagation. PeerJ 5:e3035. doi:10.7717/peerj.3035. 28289564 PMC5345454

[12] Sieber CMK, Probst AJ, Sharrar A, Thomas BC, Hess M, Tringe SG, Banfield JF. 2018. Recovery of genomes from metagenomes via a dereplication, aggregation and scoring strategy. Nat Microbiol 3:836–843. doi:10.1038/s41564-018-0171-1. 29807988 PMC6786971

[13] Olm MR, Brown CT, Brooks B, Banfield JF. 2017. dRep: a tool for fast and accurate genomic comparisons that enables improved genome recovery from metagenomes through de-replication. ISME J 11:2864–2868. doi:10.1038/ismej.2017.126. 28742071 PMC5702732

[14] Chaumeil PA, Mussig AJ, Hugenholtz P, Parks DH. 2019. GTDB-Tk: a toolkit to classify genomes with the Genome Taxonomy Database. Bioinformatics 36:1925–1927. doi:10.1093/bioinformatics/btz848. 31730192 PMC7703759

[15] Simpson JT, Wong K, Jackman SD, Schein JE, Jones SJ, Birol I. 2009. ABySS: a parallel assembler for short read sequence data. Genome Res 19:1117–1123. doi:10.1101/gr.089532.108. 19251739 PMC2694472

[16] Tang S, Gong Y, Edwards EA. 2012. Semi-automatic in silico gap closure enabled de novo assembly of two Dehalobacter genomes from metagenomic data. PLoS One 7:e52038. doi:10.1371/journal.pone.0052038. 23284863 PMC3528712

[17] Kearse M, Moir R, Wilson A, Stones-Havas S, Cheung M, Sturrock S, Buxton S, Cooper A, Markowitz S, Duran C, Thierer T, Ashton B, Meintjes P, Drummond A. 2012. Geneious Basic: an integrated and extendable desktop software platform for the organization and analysis of sequence data. Bioinformatics 28:1647–1649. doi:10.1093/bioinformatics/bts199. 22543367 PMC3371832

[18] Parks DH, Imelfort M, Skennerton CT, Hugenholtz P, Tyson GW. 2015. CheckM: assessing the quality of microbial genomes recovered from isolates, single cells, and metagenomes. Genome Res 25:1043–1055. doi:10.1101/gr.186072.114. 25977477 PMC4484387

[19] Eren AM, Kiefl E, Shaiber A, Veseli I, Miller SE, Schechter MS, Fink I, Pan JN, Yousef M, Fogarty EC, Trigodet F, Watson AR, Esen ÖC, Moore RM, Clayssen Q, Lee MD, Kivenson V, Graham ED, Merrill BD, Karkman A, Blankenberg D, Eppley JM, Sjödin A, Scott JJ, Vázquez-Campos X, McKay LJ, McDaniel EA, Stevens SLR, Anderson RE, Fuessel J, Fernandez-Guerra A, Maignien L, Delmont TO, Willis AD. 2021. Community-led, integrated, reproducible multi-omics with anvi'o. Nat Microbiol 6:3–6. doi:10.1038/s41564-020-00834-3. 33349678 PMC8116326

[20] Chen X, Molenda O, Brown CT, Toth CRA, Guo S, Luo F, Howe J, Nesbø CL, He C, Montabana EA, Cate JHD, Banfield JF, Edwards EA. 2023. “*Candidatus* Nealsonbacteria” are likely biomass recycling ectosymbionts of methanogenic archaea in a stable benzene-degrading enrichment culture. Appl Environ Microbiol 89:e00025-23. doi:10.1128/aem.00025-23. PMC10231131 37098974

